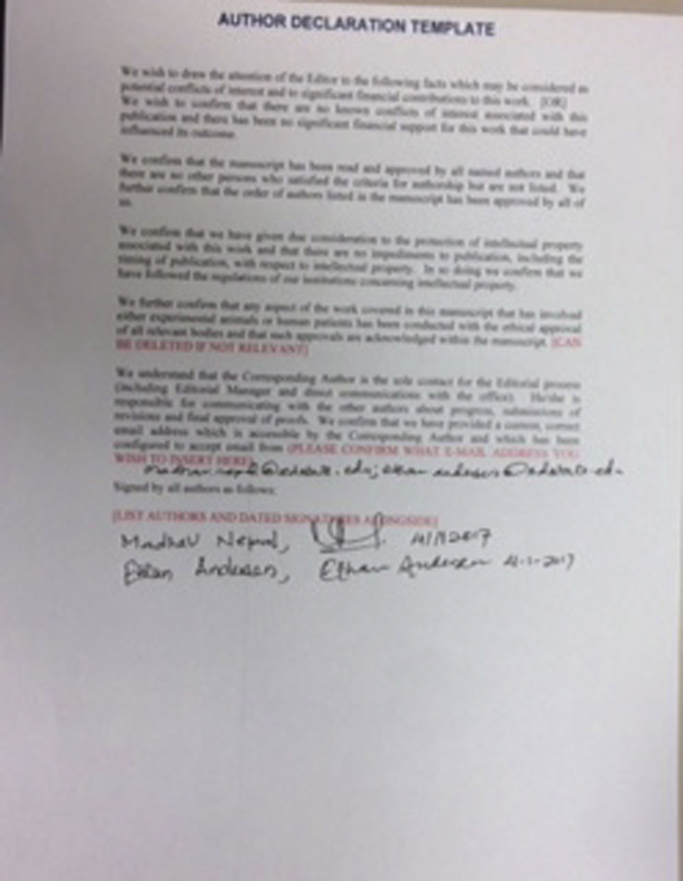# Data on the genome-wide identification of CNL R-genes in *Setaria italica* (L.) P. Beauv.

**DOI:** 10.1016/j.dib.2017.05.035

**Published:** 2017-05-30

**Authors:** Ethan J. Andersen, Madhav P. Nepal

**Affiliations:** Department of Biology and Microbiology, South Dakota State University, Brookings, SD, USA

**Keywords:** Foxtail millet, Gene duplication, NBS-LRR, Pathogen resistance, Purifying selection, Synteny

## Abstract

We report data associated with the identification of 242 disease resistance genes (R-genes) in the genome of *Setaria italica* as presented in “Genetic diversity of disease resistance genes in foxtail millet (*Setaria italica* L.)” (Andersen and Nepal, 2017) [1]. Our data describe the structure and evolution of the Coiled-coil, Nucleotide-binding site, Leucine-rich repeat (CNL) R-genes in foxtail millet. The CNL genes were identified through rigorous extraction and analysis of recently available plant genome sequences using cutting-edge analytical software. Data visualization includes gene structure diagrams, chromosomal syntenic maps, a chromosomal density plot, and a maximum-likelihood phylogenetic tree comparing *Sorghum bicolor*, *Panicum virgatum*, *Setaria italica*, and *Arabidopsis thaliana*. Compilation of InterProScan annotations, Gene Ontology (GO) annotations, and Basic Local Alignment Search Tool (BLAST) results for the 242 R-genes identified in the foxtail millet genome are also included in tabular format.

**Specifications Table**

**Table:** 

Subject area	Genomics, Bioinformatics
More specific subject area	Disease resistance in crops
Type of data	Figures and Tables
How data was acquired	Bioinformatics analysis of genomic data
Data format	Filtered and analyzed
Experimental factors	All methods were carried out *in silico* using bioinformatics software.
Experimental features	R-gene sequences and annotations were compiled and analyzed.
Data source location	Brookings, South Dakota, USA; sequences were retrieved from cited databases (see Experimental design, materials and methods).
Data accessibility	Data are with this article.

**Value of the data**

• Gene structure and homology data elucidate splicing patterns and protein function, establishing a basis for functional characterization.
• Genomic synteny and phylogeny among crops illustrate evolutionary divergence that may be useful for the production of transgenic varieties aimed at the conferral of pathogen resistance.
• Phylogeny provides insight into the evolution of pathogen resistance through R-gene retention, loss, and diversification**.**

## 1 Data

**Fig. 1:**
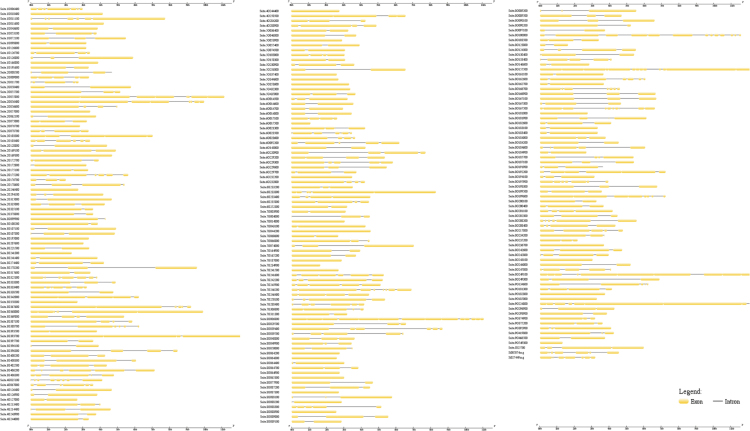
Exon variation across foxtail millet CNL gene sequences, with exons and introns represented by yellow bars and grey lines, respectively.

**Fig. 2:**
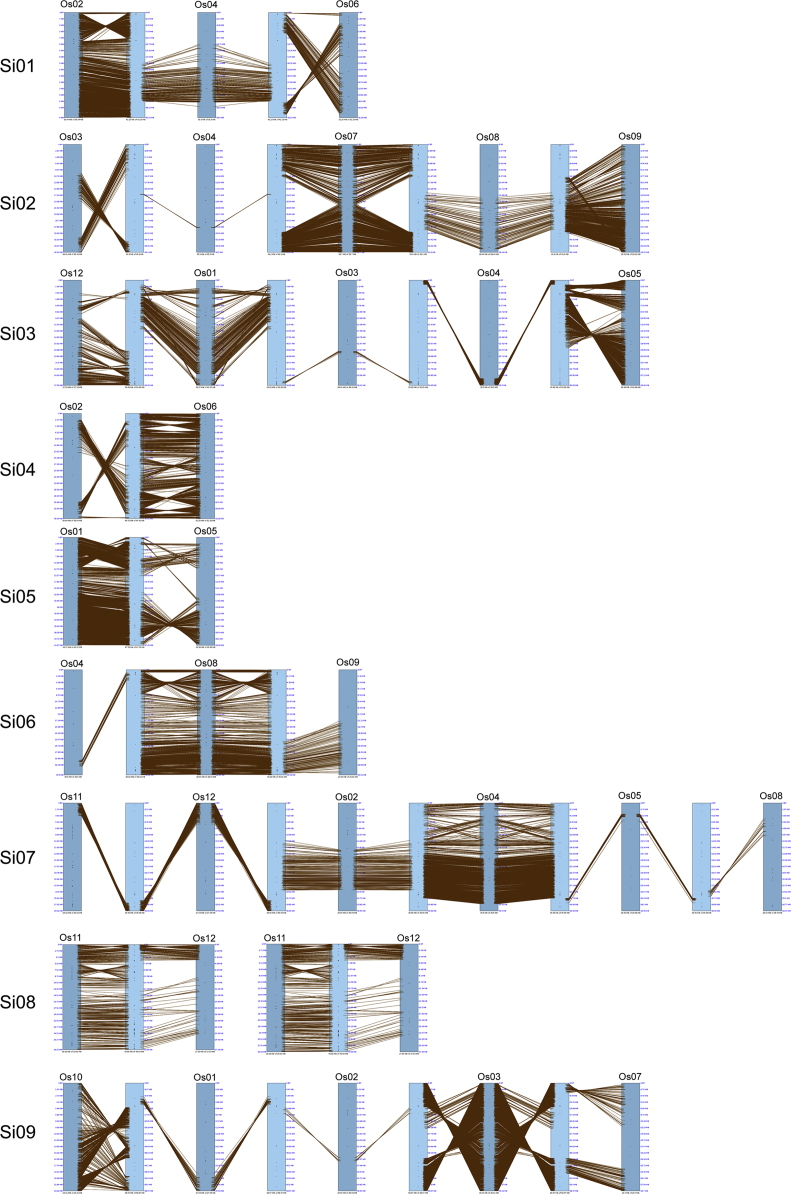
Synteny between selected foxtail millet and rice chromosomes, illustrating orthologous relationships of CNL genes. Comparisons are shown for substantial chromosomal inversions and duplications. Maps are arranged by foxtail millet chromosomes (Si) in rows, with rice chromosomes (Os) being individually labeled.

**Fig. 3:**
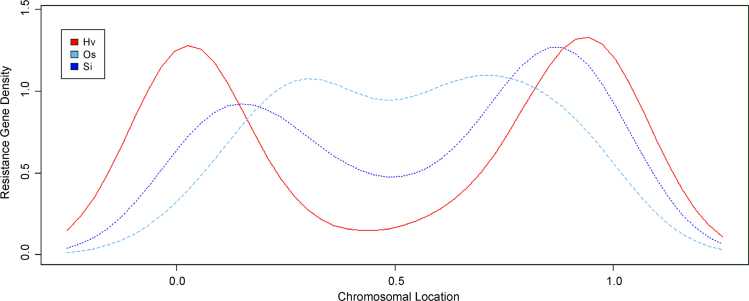
Density plot of chromosomal R-gene locations for *H. vulgare*, *O. sativa*, and *S. italica*, labeled as Hv, Os, and Si, respectively.

**Fig. 4:**
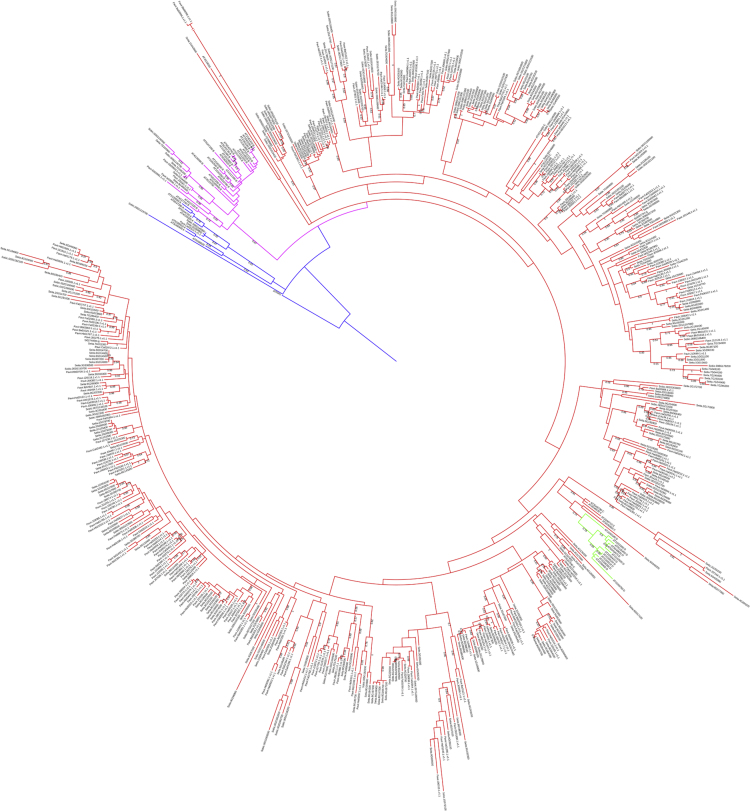
Maximum-likelihood phylogenetic analysis of the NB-ARC amino acid sequences of R-genes in *Setaria italica* (Seita), *Sorghum bicolor* (Sobic), *Panicum virgatum* (Pavir), and *Arabidopsis thaliana* (AT) using the JTT+G+F model and 100 bootstrap replicates, rooted on the outgroup p25941 from *Streptomyces coelicolor*. CNL clades A, B, C, and D are shown in blue, pink, red, and green, respectively.

These data provide detailed information regarding foxtail millet CNL R-genes [1]. Generated from sequence annotations, gene structure diagrams for each of the 242 genes were compiled (Fig. 1), along with InterProScan and Gene Ontology (GO) annotations (Table 1). Utilizing CNL R-gene genomic locations, chromosomal syntenic maps (Fig. 2) and R-gene density (Fig. 3) are displayed. Based on R-protein sequences, homologs were compiled (Table 2) and a maximum-likelihood phylogenetic tree (Fig. 4) displays the evolutionary relationships between *Setaria italica*, *Arabidopsis thaliana*, *Sorghum bicolor*, and *Panicum virgatum* accessions.

**Table 1:** InterProScan and GO annotation identities and descriptions for the foxtail millet CNL sequences, accessed from the Biomart function of Ensembl Genomes.

Annotation Name:	Annotation ID:	Accessions:									
AAA+ ATPase domain	IPR003593	Seita.7G250100	Seita.6G014500	Seita.6G014700	Seita.2G315000	Seita.5G158300	Seita.3G387100	Seita.8G039400	Seita.7G164900	Seita.8G087200	Seita.6G014600
Armadillo-like helical	IPR011989	Seita.1G043600									
Armadillo-type fold	IPR016024	Seita.1G043600									
DNA-binding WRKY	IPR003657	Seita.2G175200									
FNIP	IPR008615	Seita.8G242600									
Leucine-rich repeat	IPR001611, IPR032675, IPR006553, and IPR003591	Seita.8G029500	Si027449m.g	Seita.8G208200	Seita.4G244000	Seita.6G233400	Seita.9G134600	Seita.3G241300	Seita.9G182800	Seita.3G278200	Seita.8G103500
		Seita.9G224800	Seita.8G048800	Seita.2G178600	Seita.6G233300	Seita.7G311300	Seita.6G232200	Seita.5G344600	Seita.2G062100	Seita.6G232800	Seita.7G164900
		Seita.2G172700	Seita.7G165200	Seita.8G133400	Seita.8G191900	Seita.7G074000	Seita.9G466500	Seita.8G100000	Seita.6G235800	Seita.2G075000	Seita.3G393500
		Seita.8G185700	Seita.3G333400	Seita.6G229700	Seita.1G126700	Seita.8G217800	Seita.7G288400	Seita.5G046800	Seita.8G161100	Seita.9G375200	Seita.2G103300
		Seita.8G089100	Seita.6G023500	Seita.3G342000	Seita.2G078700	Seita.5G036400	Seita.7G250100	Seita.6G023600	Seita.7G066800	Seita.8G088100	Seita.4G284200
		Seita.8G181000	Seita.1G072100	Seita.7G060600	Seita.8G124300	Seita.8G184200	Si005074m.g	Seita.8G064700	Seita.3G107100	Seita.3G369800	Seita.3G395700
		Seita.4G126400	Seita.8G039500	Seita.7G245900	Seita.2G169100	Seita.8G183100	Seita.8G249100	Seita.6G229300	Seita.3G195000	Seita.8G200100	Seita.7G306800
		Seita.8G167500	Seita.8G236700	Seita.2G172800	Seita.8G087300	Seita.4G250500	Seita.3G274400	Seita.8G187100	Seita.3G207600	Seita.8G202300	Seita.8G184000
		Seita.8G166700	Seita.5G074500	Seita.6G252300	Seita.9G392900	Seita.8G199600	Seita.3G400300	Seita.8G201100	Seita.5G337400	Seita.3G367600	Seita.7G043100
		Seita.8G064300	Seita.4G213400	Seita.2G378800	Seita.1G166800	Seita.8G162600	Seita.3G325300	Seita.6G014500	Seita.3G350300	Seita.3G400200	Seita.6G220900
		Seita.7G044200	Seita.7G014800	Seita.5G053900	Seita.1G053100	Seita.2G128800	Seita.8G088200	Seita.2G103400	Seita.7G241600	Seita.8G146600	Seita.7G241500
		Seita.7G242200	Seita.5G055400	Seita.2G055800	Seita.6G014700	Seita.9G296900	Seita.3G338500	Seita.2G008500	Seita.1G098800	Seita.8G181900	Seita.2G246400
		Seita.1G191600	Seita.8G247800	Seita.2G175100	Seita.2G076700	Seita.8G182600	Seita.8G050000	Seita.3G406300	Seita.3G393700	Seita.8G184900	Seita.8G090200
		Seita.7G246200	Seita.3G100200	Seita.2G335000	Seita.8G199500	Seita.8G130400	Seita.4G214400	Seita.9G374900	Seita.3G241400	Seita.2G315000	Seita.3G107300
		Seita.5G353600	Seita.8G244000	Seita.9G298900	Seita.6G021300	Seita.3G396300	Seita.4G288900	Seita.2G009000	Seita.8G234200	Seita.5G158300	Seita.7G246400
		Seita.4G067000	Seita.2G011700	Seita.2G175200	Seita.2G294100	Seita.6G017100	Seita.9G181300	Seita.2G056400	Seita.8G089500	Seita.7G003900	Seita.1G006400
		Seita.8G208400	Seita.3G368000	Seita.8G242800	Seita.2G055500	Seita.4G126900	Seita.8G194100	Seita.1G126800	Seita.5G231000	Seita.6G014800	Seita.8G235200
		Seita.2G050400	Seita.8G249300	Seita.8G242600	Seita.6G229600	Seita.3G387100	Seita.6G229200	Seita.3G221500	Seita.8G198300	Seita.8G192200	Seita.8G039400
		Seita.8G184600	Seita.8G090100	Seita.8G243100	Seita.8G195900	Seita.J025700	Seita.8G065300	Seita.8G167100	Seita.8G006800	Seita.1G126600	Seita.8G200400
		Seita.8G064200	Seita.8G049900	Seita.3G406200	Seita.6G233200	Seita.5G103000	Seita.8G162700	Seita.3G388700	Seita.8G064900	Seita.8G087200	Seita.8G183400
		Seita.3G317600	Seita.8G089000	Seita.5G230900	Seita.1G011600	Seita.2G056600	Seita.5G435000	Seita.3G333300	Seita.6G092200	Seita.2G057000	Seita.1G011100
		Seita.8G089200	Seita.3G402500	Seita.4G243900	Seita.8G088300	Seita.8G064400	Seita.8G077900	Seita.1G010400	Seita.5G432300	Seita.4G244400	Seita.9G185000
		Seita.6G014600	Seita.3G099900	Seita.2G169300	Seita.8G166900	Seita.7G187000	Seita.8G155500	Seita.8G167300	Seita.6G143000	Seita.8G088900	Seita.4G127000
		Seita.2G335100	Seita.9G419800								
Mannose-binding lectin	IPR001229	Seita.8G124300									
NB-ARC	IPR002182	Seita.2G075000	Seita.3G393500	Seita.8G185700	Seita.3G333400	Seita.6G229700	Seita.8G029500	Seita.1G126700	Seita.8G217800	Seita.7G288400	Si027449m.g
		Seita.5G046800	Seita.8G208200	Seita.8G161100	Seita.9G375200	Seita.2G103300	Seita.8G089100	Seita.6G023500	Seita.3G342000	Seita.2G078700	Seita.5G036400
		Seita.9G549300	Seita.7G250100	Seita.2G176700	Seita.6G023600	Seita.7G066800	Seita.8G088100	Seita.4G284200	Seita.8G181000	Seita.1G072100	Seita.7G060600
		Seita.8G124300	Seita.4G244000	Seita.8G184200	Si005074m.g	Seita.8G064700	Seita.3G107100	Seita.3G369800	Seita.3G395700	Seita.4G126400	Seita.8G039500
		Seita.7G245900	Seita.2G169100	Seita.8G183100	Seita.8G249100	Seita.6G229300	Seita.3G195000	Seita.8G200100	Seita.7G306800	Seita.8G167500	Seita.8G236700
		Seita.7G234900	Seita.2G172800	Seita.8G087300	Seita.4G250500	Seita.3G396100	Seita.3G274400	Seita.8G187100	Seita.3G207600	Seita.8G202300	Seita.6G233400
		Seita.8G184000	Seita.8G166700	Seita.5G074500	Seita.6G252300	Seita.9G392900	Seita.8G199600	Seita.3G400300	Seita.8G201100	Seita.5G337400	Seita.3G367600
		Seita.7G043100	Seita.8G064300	Seita.4G213400	Seita.2G378800	Seita.1G166800	Seita.8G162600	Seita.3G325300	Seita.6G014500	Seita.3G350300	Seita.3G400200
		Seita.6G220900	Seita.7G044200	Seita.7G014800	Seita.5G053900	Seita.1G053100	Seita.8G097100	Seita.2G128800	Seita.8G088200	Seita.2G103400	Seita.7G241600
		Seita.9G134600	Seita.8G146600	Seita.7G241500	Seita.7G242200	Seita.5G055400	Seita.2G055800	Seita.6G014700	Seita.9G296900	Seita.3G338500	Seita.3G241300
		Seita.2G008500	Seita.4G035100	Seita.1G098800	Seita.9G182800	Seita.8G181900	Seita.2G246400	Seita.1G191600	Seita.8G247800	Seita.2G175100	Seita.2G076700
		Seita.8G182600	Seita.3G278200	Seita.8G103500	Seita.8G050000	Seita.3G406300	Seita.3G393700	Seita.8G184900	Seita.8G090200	Seita.7G246200	Seita.3G100200
		Seita.2G335000	Seita.8G199500	Seita.8G130400	Seita.4G214400	Seita.9G374900	Seita.3G241400	Seita.2G315000	Seita.3G107300	Seita.5G353600	Seita.8G244000
		Seita.9G298900	Seita.9G224800	Seita.6G021300	Seita.3G396300	Seita.4G288900	Seita.2G009000	Seita.8G234200	Seita.5G158300	Seita.7G246400	Seita.4G067000
		Seita.8G123800	Seita.2G011700	Seita.8G048800	Seita.2G175200	Seita.2G294100	Seita.6G017100	Seita.9G181300	Seita.2G056400	Seita.8G089500	Seita.7G003900
		Seita.1G006400	Seita.8G208400	Seita.3G368000	Seita.8G242800	Seita.2G055500	Seita.4G126900	Seita.2G178600	Seita.8G194100	Seita.6G233300	Seita.1G126800
		Seita.5G231000	Seita.6G014800	Seita.8G235200	Seita.2G050400	Seita.8G249300	Seita.8G242600	Seita.6G229600	Seita.7G311300	Seita.3G387100	Seita.6G229200
		Seita.3G221500	Seita.6G232200	Seita.8G198300	Seita.8G192200	Seita.5G344600	Seita.8G039400	Seita.8G184600	Seita.8G090100	Seita.8G243100	Seita.2G062100
		Seita.8G195900	Seita.6G232800	Seita.7G164900	Seita.J025700	Seita.8G065300	Seita.8G167100	Seita.8G006800	Seita.1G126600	Seita.8G200400	Seita.8G064200
		Seita.8G049900	Seita.3G406200	Seita.6G233200	Seita.5G103000	Seita.8G162700	Seita.3G388700	Seita.8G064900	Seita.8G087200	Seita.2G172700	Seita.8G183400
		Seita.3G317600	Seita.7G165200	Seita.8G089000	Seita.5G230900	Seita.8G133400	Seita.1G011600	Seita.2G056600	Seita.1G043600	Seita.5G435000	Seita.3G333300
		Seita.6G092200	Seita.2G057000	Seita.1G011100	Seita.8G089200	Seita.8G191900	Seita.3G402500	Seita.7G074000	Seita.4G243900	Seita.8G088300	Seita.9G466500
		Seita.8G064400	Seita.8G077900	Seita.1G010400	Seita.5G432300	Seita.4G244400	Seita.9G185000	Seita.6G014600	Seita.3G099900	Seita.2G169300	Seita.8G100000
		Seita.8G166900	Seita.7G187000	Seita.8G155500	Seita.7G004000	Seita.8G167300	Seita.6G235800	Seita.6G143000	Seita.8G088900	Seita.4G127000	Seita.2G335100
		Seita.6G017500	Seita.9G419800								
No apical meristem (NAM) protein	IPR003441	Seita.8G039400									
Phospholipase C, phosphatidylinositol-specific, Y domain	IPR001711	Seita.3G100200									
P-loop containing nucleoside triphosphate hydrolase	IPR027417	Seita.2G075000	Seita.3G393500	Seita.8G185700	Seita.3G333400	Seita.6G229700	Seita.8G029500	Seita.1G126700	Seita.8G217800	Seita.7G288400	Si027449m.g
		Seita.5G046800	Seita.8G208200	Seita.8G161100	Seita.9G375200	Seita.2G103300	Seita.8G089100	Seita.6G023500	Seita.3G342000	Seita.2G078700	Seita.5G036400
		Seita.9G549300	Seita.7G250100	Seita.2G176700	Seita.6G023600	Seita.7G066800	Seita.8G088100	Seita.4G284200	Seita.8G181000	Seita.1G072100	Seita.7G060600
		Seita.8G124300	Seita.4G244000	Seita.8G184200	Si005074m.g	Seita.8G064700	Seita.3G107100	Seita.3G369800	Seita.3G395700	Seita.4G126400	Seita.8G039500
		Seita.7G245900	Seita.2G169100	Seita.8G183100	Seita.8G249100	Seita.6G229300	Seita.3G195000	Seita.8G200100	Seita.7G306800	Seita.8G167500	Seita.8G236700
		Seita.7G234900	Seita.2G172800	Seita.8G087300	Seita.4G250500	Seita.3G396100	Seita.3G274400	Seita.8G187100	Seita.3G207600	Seita.8G202300	Seita.6G233400
		Seita.8G184000	Seita.8G166700	Seita.5G074500	Seita.6G252300	Seita.9G392900	Seita.8G199600	Seita.3G400300	Seita.8G201100	Seita.5G337400	Seita.3G367600
		Seita.7G043100	Seita.8G064300	Seita.4G213400	Seita.2G378800	Seita.1G166800	Seita.8G162600	Seita.3G325300	Seita.6G014500	Seita.3G350300	Seita.3G400200
		Seita.6G220900	Seita.7G044200	Seita.7G014800	Seita.5G053900	Seita.1G053100	Seita.8G097100	Seita.2G128800	Seita.8G088200	Seita.2G103400	Seita.7G241600
		Seita.9G134600	Seita.8G146600	Seita.7G241500	Seita.7G242200	Seita.5G055400	Seita.2G055800	Seita.6G014700	Seita.9G296900	Seita.3G338500	Seita.3G241300
		Seita.2G008500	Seita.4G035100	Seita.1G098800	Seita.9G182800	Seita.8G181900	Seita.2G246400	Seita.1G191600	Seita.8G247800	Seita.2G175100	Seita.2G076700
		Seita.8G182600	Seita.3G278200	Seita.8G103500	Seita.8G050000	Seita.3G406300	Seita.3G393700	Seita.8G184900	Seita.8G090200	Seita.7G246200	Seita.3G100200
		Seita.2G335000	Seita.8G199500	Seita.8G130400	Seita.4G214400	Seita.9G374900	Seita.3G241400	Seita.2G315000	Seita.3G107300	Seita.5G353600	Seita.8G244000
		Seita.9G298900	Seita.9G224800	Seita.6G021300	Seita.3G396300	Seita.4G288900	Seita.2G009000	Seita.8G234200	Seita.5G158300	Seita.7G246400	Seita.4G067000
		Seita.8G123800	Seita.2G011700	Seita.8G048800	Seita.2G175200	Seita.2G294100	Seita.6G017100	Seita.9G181300	Seita.2G056400	Seita.8G089500	Seita.7G003900
		Seita.1G006400	Seita.8G208400	Seita.3G368000	Seita.8G242800	Seita.2G055500	Seita.4G126900	Seita.2G178600	Seita.8G194100	Seita.6G233300	Seita.1G126800
		Seita.5G231000	Seita.6G014800	Seita.8G235200	Seita.2G050400	Seita.8G249300	Seita.8G242600	Seita.6G229600	Seita.7G311300	Seita.3G387100	Seita.6G229200
		Seita.3G221500	Seita.6G232200	Seita.8G198300	Seita.8G192200	Seita.5G344600	Seita.8G039400	Seita.8G184600	Seita.8G090100	Seita.8G243100	Seita.2G062100
		Seita.8G195900	Seita.6G232800	Seita.7G164900	Seita.J025700	Seita.8G065300	Seita.8G167100	Seita.8G006800	Seita.1G126600	Seita.8G200400	Seita.8G064200
		Seita.8G049900	Seita.3G406200	Seita.6G233200	Seita.5G103000	Seita.8G162700	Seita.3G388700	Seita.8G064900	Seita.8G087200	Seita.2G172700	Seita.8G183400
		Seita.3G317600	Seita.7G165200	Seita.8G089000	Seita.5G230900	Seita.8G133400	Seita.1G011600	Seita.2G056600	Seita.1G043600	Seita.5G435000	Seita.3G333300
		Seita.6G092200	Seita.2G057000	Seita.1G011100	Seita.8G089200	Seita.8G191900	Seita.3G402500	Seita.7G074000	Seita.4G243900	Seita.8G088300	Seita.9G466500
		Seita.8G064400	Seita.8G077900	Seita.1G010400	Seita.5G432300	Seita.4G244400	Seita.9G185000	Seita.6G014600	Seita.3G099900	Seita.2G169300	Seita.8G100000
		Seita.8G166900	Seita.7G187000	Seita.8G155500	Seita.7G004000	Seita.8G167300	Seita.6G235800	Seita.6G143000	Seita.8G088900	Seita.4G127000	Seita.2G335100
		Seita.6G017500	Seita.9G419800								
Powdery mildew resistance protein, RPW8 domain	IPR008808	Seita.3G369800									
Protein kinase, ATP binding site	IPR017441	Seita.4G035100	Seita.8G100000								
Protein kinase, catalytic domain	IPR000719	Seita.4G035100	Seita.8G100000								
Protein kinase-like domain	IPR011009	Seita.4G035100	Seita.8G100000								
Serine/threonine- / dual specificity protein kinase, catalytic domain	IPR002290	Seita.4G035100	Seita.8G100000								
Serine/threonine-protein kinase, active site	IPR008271	Seita.4G035100	Seita.8G100000								
Tetratricopeptide repeat	IPR019734	Seita.8G133400									
Zinc finger, BED-type predicted	IPR003656	Seita.7G242200	Seita.J025700								
Zinc finger, C2H2	IPR007087	Seita.1G053100									
ADP binding	GO:0043531	Seita.7G250100	Seita.6G014500	Seita.6G014700	Seita.2G315000	Seita.5G158300	Seita.3G387100	Seita.8G039400	Seita.7G164900	Seita.8G087200	Seita.6G014600
		Seita.1G043600	Seita.2G175200	Seita.8G242600	Seita.8G029500	Si027449m.g	Seita.8G208200	Seita.4G244000	Seita.6G233400	Seita.9G134600	Seita.3G241300
		Seita.9G182800	Seita.3G278200	Seita.8G103500	Seita.9G224800	Seita.8G048800	Seita.2G178600	Seita.6G233300	Seita.7G311300	Seita.6G232200	Seita.5G344600
		Seita.2G062100	Seita.6G232800	Seita.2G172700	Seita.7G165200	Seita.8G133400	Seita.8G191900	Seita.7G074000	Seita.9G466500	Seita.8G100000	Seita.6G235800
		Seita.2G075000	Seita.3G393500	Seita.8G185700	Seita.3G333400	Seita.6G229700	Seita.1G126700	Seita.8G217800	Seita.7G288400	Seita.5G046800	Seita.8G161100
		Seita.9G375200	Seita.2G103300	Seita.8G089100	Seita.6G023500	Seita.3G342000	Seita.2G078700	Seita.5G036400	Seita.6G023600	Seita.7G066800	Seita.8G088100
		Seita.4G284200	Seita.8G181000	Seita.1G072100	Seita.7G060600	Seita.8G124300	Seita.8G184200	Si005074m.g	Seita.8G064700	Seita.3G107100	Seita.3G369800
		Seita.3G395700	Seita.4G126400	Seita.8G039500	Seita.7G245900	Seita.2G169100	Seita.8G183100	Seita.8G249100	Seita.6G229300	Seita.3G195000	Seita.8G200100
		Seita.7G306800	Seita.8G167500	Seita.8G236700	Seita.2G172800	Seita.8G087300	Seita.4G250500	Seita.3G274400	Seita.8G187100	Seita.3G207600	Seita.8G202300
		Seita.8G184000	Seita.8G166700	Seita.5G074500	Seita.6G252300	Seita.9G392900	Seita.8G199600	Seita.3G400300	Seita.8G201100	Seita.5G337400	Seita.3G367600
		Seita.7G043100	Seita.8G064300	Seita.4G213400	Seita.2G378800	Seita.1G166800	Seita.8G162600	Seita.3G325300	Seita.3G350300	Seita.3G400200	Seita.6G220900
		Seita.7G044200	Seita.7G014800	Seita.5G053900	Seita.1G053100	Seita.2G128800	Seita.8G088200	Seita.2G103400	Seita.7G241600	Seita.8G146600	Seita.7G241500
		Seita.7G242200	Seita.5G055400	Seita.2G055800	Seita.9G296900	Seita.3G338500	Seita.2G008500	Seita.1G098800	Seita.8G181900	Seita.2G246400	Seita.1G191600
		Seita.8G247800	Seita.2G175100	Seita.2G076700	Seita.8G182600	Seita.8G050000	Seita.3G406300	Seita.3G393700	Seita.8G184900	Seita.8G090200	Seita.7G246200
		Seita.3G100200	Seita.2G335000	Seita.8G199500	Seita.8G130400	Seita.4G214400	Seita.9G374900	Seita.3G241400	Seita.3G107300	Seita.5G353600	Seita.8G244000
		Seita.9G298900	Seita.6G021300	Seita.3G396300	Seita.4G288900	Seita.2G009000	Seita.8G234200	Seita.7G246400	Seita.4G067000	Seita.2G011700	Seita.2G294100
		Seita.6G017100	Seita.9G181300	Seita.2G056400	Seita.8G089500	Seita.7G003900	Seita.1G006400	Seita.8G208400	Seita.3G368000	Seita.8G242800	Seita.2G055500
		Seita.4G126900	Seita.8G194100	Seita.1G126800	Seita.5G231000	Seita.6G014800	Seita.8G235200	Seita.2G050400	Seita.8G249300	Seita.6G229600	Seita.6G229200
		Seita.3G221500	Seita.8G198300	Seita.8G192200	Seita.8G184600	Seita.8G090100	Seita.8G243100	Seita.8G195900	Seita.J025700	Seita.8G065300	Seita.8G167100
		Seita.8G006800	Seita.1G126600	Seita.8G200400	Seita.8G064200	Seita.8G049900	Seita.3G406200	Seita.6G233200	Seita.5G103000	Seita.8G162700	Seita.3G388700
		Seita.8G064900	Seita.8G183400	Seita.3G317600	Seita.8G089000	Seita.5G230900	Seita.1G011600	Seita.2G056600	Seita.5G435000	Seita.3G333300	Seita.6G092200
		Seita.2G057000	Seita.1G011100	Seita.8G089200	Seita.3G402500	Seita.4G243900	Seita.8G088300	Seita.8G064400	Seita.8G077900	Seita.1G010400	Seita.5G432300
		Seita.4G244400	Seita.9G185000	Seita.3G099900	Seita.2G169300	Seita.8G166900	Seita.7G187000	Seita.8G155500	Seita.8G167300	Seita.6G143000	Seita.8G088900
		Seita.4G127000	Seita.2G335100	Seita.9G419800	Seita.9G549300	Seita.2G176700	Seita.7G234900	Seita.3G396100	Seita.8G097100	Seita.4G035100	Seita.8G123800
		Seita.7G004000	Seita.6G017500								
ATP binding	GO:0005524	Seita.7G250100	Seita.6G014500	Seita.6G014700	Seita.2G315000	Seita.5G158300	Seita.3G387100	Seita.8G039400	Seita.7G164900	Seita.8G087200	Seita.6G014600
		Seita.8G100000	Seita.8G039500	Seita.8G184000	Seita.7G246200	Seita.6G021300	Seita.2G009000	Seita.7G246400	Seita.6G014800	Seita.2G176700	Seita.4G035100
Binding	GO:0005488	Seita.1G043600									
Defense response	GO:0006952	Seita.7G250100	Seita.6G014500	Seita.6G014700	Seita.2G315000	Seita.5G158300	Seita.3G387100	Seita.8G039400	Seita.7G164900	Seita.8G087200	Seita.6G014600
		Seita.1G043600	Seita.2G175200	Seita.8G242600	Seita.8G029500	Si027449m.g	Seita.8G208200	Seita.4G244000	Seita.6G233400	Seita.9G134600	Seita.3G241300
		Seita.9G182800	Seita.3G278200	Seita.8G103500	Seita.9G224800	Seita.8G048800	Seita.2G178600	Seita.6G233300	Seita.7G311300	Seita.6G232200	Seita.5G344600
		Seita.2G062100	Seita.6G232800	Seita.2G172700	Seita.7G165200	Seita.8G133400	Seita.8G191900	Seita.7G074000	Seita.9G466500	Seita.8G100000	Seita.6G235800
		Seita.2G075000	Seita.3G393500	Seita.8G185700	Seita.3G333400	Seita.6G229700	Seita.1G126700	Seita.8G217800	Seita.7G288400	Seita.5G046800	Seita.8G161100
		Seita.9G375200	Seita.2G103300	Seita.8G089100	Seita.6G023500	Seita.3G342000	Seita.2G078700	Seita.5G036400	Seita.6G023600	Seita.7G066800	Seita.8G088100
		Seita.4G284200	Seita.8G181000	Seita.1G072100	Seita.7G060600	Seita.8G124300	Seita.8G184200	Si005074m.g	Seita.8G064700	Seita.3G107100	Seita.3G369800
		Seita.3G395700	Seita.4G126400	Seita.8G039500	Seita.7G245900	Seita.2G169100	Seita.8G183100	Seita.8G249100	Seita.6G229300	Seita.3G195000	Seita.8G200100
		Seita.7G306800	Seita.8G167500	Seita.8G236700	Seita.2G172800	Seita.8G087300	Seita.4G250500	Seita.3G274400	Seita.8G187100	Seita.3G207600	Seita.8G202300
		Seita.8G184000	Seita.8G166700	Seita.5G074500	Seita.6G252300	Seita.9G392900	Seita.8G199600	Seita.3G400300	Seita.8G201100	Seita.5G337400	Seita.3G367600
		Seita.7G043100	Seita.8G064300	Seita.4G213400	Seita.2G378800	Seita.1G166800	Seita.8G162600	Seita.3G325300	Seita.3G350300	Seita.3G400200	Seita.6G220900
		Seita.7G044200	Seita.7G014800	Seita.5G053900	Seita.1G053100	Seita.2G128800	Seita.8G088200	Seita.2G103400	Seita.7G241600	Seita.8G146600	Seita.7G241500
		Seita.7G242200	Seita.5G055400	Seita.2G055800	Seita.9G296900	Seita.3G338500	Seita.2G008500	Seita.1G098800	Seita.8G181900	Seita.2G246400	Seita.1G191600
		Seita.8G247800	Seita.2G175100	Seita.2G076700	Seita.8G182600	Seita.8G050000	Seita.3G406300	Seita.3G393700	Seita.8G184900	Seita.8G090200	Seita.7G246200
		Seita.3G100200	Seita.2G335000	Seita.8G199500	Seita.8G130400	Seita.4G214400	Seita.9G374900	Seita.3G241400	Seita.3G107300	Seita.5G353600	Seita.8G244000
		Seita.9G298900	Seita.6G021300	Seita.3G396300	Seita.4G288900	Seita.2G009000	Seita.8G234200	Seita.7G246400	Seita.4G067000	Seita.2G011700	Seita.2G294100
		Seita.6G017100	Seita.9G181300	Seita.2G056400	Seita.8G089500	Seita.7G003900	Seita.1G006400	Seita.8G208400	Seita.3G368000	Seita.8G242800	Seita.2G055500
		Seita.4G126900	Seita.8G194100	Seita.1G126800	Seita.5G231000	Seita.6G014800	Seita.8G235200	Seita.2G050400	Seita.8G249300	Seita.6G229600	Seita.6G229200
		Seita.3G221500	Seita.8G198300	Seita.8G192200	Seita.8G184600	Seita.8G090100	Seita.8G243100	Seita.8G195900	Seita.J025700	Seita.8G065300	Seita.8G167100
		Seita.8G006800	Seita.1G126600	Seita.8G200400	Seita.8G064200	Seita.8G049900	Seita.3G406200	Seita.6G233200	Seita.5G103000	Seita.8G162700	Seita.3G388700
		Seita.8G064900	Seita.8G183400	Seita.3G317600	Seita.8G089000	Seita.5G230900	Seita.1G011600	Seita.2G056600	Seita.5G435000	Seita.3G333300	Seita.6G092200
		Seita.2G057000	Seita.1G011100	Seita.8G089200	Seita.3G402500	Seita.4G243900	Seita.8G088300	Seita.8G064400	Seita.8G077900	Seita.1G010400	Seita.5G432300
		Seita.4G244400	Seita.9G185000	Seita.3G099900	Seita.2G169300	Seita.8G166900	Seita.7G187000	Seita.8G155500	Seita.8G167300	Seita.6G143000	Seita.8G088900
		Seita.4G127000	Seita.2G335100	Seita.9G419800	Seita.9G549300	Seita.2G176700	Seita.7G234900	Seita.3G396100	Seita.8G097100	Seita.4G035100	Seita.8G123800
		Seita.7G004000	Seita.6G017500								
DNA binding	GO:0003677	Seita.8G039400	Seita.7G242200	Seita.J025700							
Intracellular signal transduction	GO:0035556	Seita.3G100200									
lipid metabolic process	GO:0006629	Seita.3G100200									
metal ion binding	GO:0046872	Seita.1G053100									
nucleoside-triphosphatase activity	GO:0017111	Seita.7G250100	Seita.6G014500	Seita.6G014700	Seita.2G315000	Seita.5G158300	Seita.3G387100	Seita.8G039400	Seita.7G164900	Seita.8G087200	Seita.6G014600
		Seita.8G039500	Seita.8G184000	Seita.7G246200	Seita.6G021300	Seita.2G009000	Seita.7G246400	Seita.6G014800	Seita.2G176700		
nucleotide binding	GO:0000166	Seita.7G250100	Seita.6G014500	Seita.6G014700	Seita.2G315000	Seita.5G158300	Seita.3G387100	Seita.8G039400	Seita.7G164900	Seita.8G087200	Seita.6G014600
		Seita.8G039500	Seita.8G184000	Seita.7G246200	Seita.6G021300	Seita.2G009000	Seita.7G246400	Seita.6G014800	Seita.2G176700	Seita.4G035100	
nucleus	GO:0005634	Seita.8G039400									
phosphatidylinositol phospholipase C activity	GO:0004435	Seita.3G100200									
protein binding	GO:0005515	Seita.7G164900	Seita.8G029500	Si027449m.g	Seita.8G208200	Seita.4G244000	Seita.6G233400	Seita.9G134600	Seita.3G241300	Seita.9G182800	Seita.3G278200
		Seita.8G103500	Seita.9G224800	Seita.8G048800	Seita.2G178600	Seita.6G233300	Seita.7G311300	Seita.6G232200	Seita.5G344600	Seita.2G062100	Seita.6G232800
		Seita.2G172700	Seita.7G165200	Seita.8G133400	Seita.8G191900	Seita.7G074000	Seita.9G466500	Seita.8G100000	Seita.6G235800		
protein kinase activity	GO:0004672	Seita.8G100000	Seita.4G035100								
protein phosphorylation	GO:0006468	Seita.8G100000	Seita.4G035100								
protein serine/threonine kinase activity	GO:0004674	Seita.8G100000	Seita.4G035100								
regulation of transcription, DNA-templated	GO:0006355	Seita.8G039400	Seita.2G175200								
sequence-specific DNA binding	GO:0043565	Seita.2G175200									
signal transduction	GO:0007165	Seita.3G100200									
transcription factor activity, sequence-specific DNA binding	GO:0003700	Seita.2G175200									
transcription, DNA-templated	GO:0006351	Seita.8G039400									
transferase activity, transferring phosphorus-containing groups	GO:0016772	Seita.8G100000	Seita.4G035100

**Table 2:** Homology generated from BLAST results for the clustered and unclustered foxtail millet sequences.

Clustered genes						Non-Clustered Genes			
Accession	Homolog	Cluster	Accession	Homolog	Cluster	Accession	Homolog	Accession	Homolog
Seita.2G008500	RPM1	2_1	Seita.7G003900	RPM1	7_1	Seita.5G055400	RGA4	Si027449m.g	RPM1
Seita.2G009000	RPM1	2_1	Seita.7G004000	RGA1	7_1	Seita.5G055400	At3g14460	Seita.8G195900	RGA4
Seita.2G103300	RPM1	2_4	Seita.7G165200	RPS2	7_2	Seita.5G055400	RGA3	Seita.8G097100	Tsn1
Seita.2G103400	RPM1	2_4	Seita.7G165200	NBS-LRR disease resistance protein homologue	7_2	Seita.5G353600	RGA3	Seita.8G130400	RDL5/RF45
Seita.2G103400	RPP13	2_4	Seita.7G165200	O1	7_2	Seita.5G036400	RPP8	Seita.8G130400	RGH1A
Seita.2G103400	RGA1	2_4	Seita.7G165200	O2	7_2	Seita.5G036400	MLA6	Seita.8G194100	Yr10/Mla1
Seita.2G103400	MLA6	2_4	Seita.7G164900	RPS2	7_2	Seita.5G435000	RPP13	Seita.8G161100	RGA1
Seita.2G172700	RGA3	2_6	Seita.7G164900	O1	7_2	Seita.5G053900	RPM1	Seita.8G161100	powdery mildew resistance protein PM3b
Seita.2G172800	RGA4	2_6	Seita.7G164900	O2	7_2	Seita.5G074500	Yr10/Mla1	Seita.8G187100	RPM1
Seita.2G172800	Pc protein A	2_6	Seita.7G242200	RGA4	7_3	Si005074m.g	Rp1	Seita.8G187100	RPP13
Seita.2G172800	Pc protein C	2_6	Seita.7G242200	B0809H07.6	7_3	Si005074m.g	Pi37	Seita.8G077900	RPP13
Seita.2G172800	pollen signalling protein with adenylyl cyclase activity-like	2_6	Seita.7G242200	XA1	7_3	Seita.5G344600	RPS2	Seita.8G198300	RPM1
Seita.2G175200	RPP13	2_7	Seita.7G241500	B0809H07.6	7_3	Seita.5G344600	PIC21	Seita.8G198300	RPP13
Seita.2G175200	WRKY transcription factor 41	2_7	Seita.7G241500	XA1	7_3	Seita.5G337400	At1g50180	Seita.2G056400	BPH14-1
Seita.2G175100	RPP13	2_7	Seita.7G241600	B0809H07.6	7_3	Seita.5G337400	RDL6/RF9	Seita.2G056400	BPH14-2
Seita.3G100200	BPH14-1	3_1	Seita.7G241600	XA1	7_3	Seita.5G337400	RPP8	Seita.2G055800	BPH14-1
Seita.3G100200	BPH14-2	3_1	Seita.7G245900	RGA4	7_4	Seita.4G288900	RPP13	Seita.2G055800	powdery mildew resistance protein PM
Seita.3G099900	BPH14-1	3_1	Seita.7G245900	NBS-LRR disease resistance protein homologue	7_4	Seita.4G288900	pollen signalling protein with adenylyl cyclase activity	Seita.2G315000	RPP13
Seita.3G099900	BPH14-2	3_1	Seita.7G246200	RGA4	7_4	Seita.4G250500	blight resistance protein B149	Seita.2G056600	BPH14-1
Seita.3G107100	powdery mildew resistance protein PM	3_2	Seita.7G246400	RGA4	7_4	Seita.4G250500	blight resistance protein RGA4	Seita.2G294100	disease resistance protein At1g50180
Seita.3G107100	BPH14-2	3_2	Seita.8G039400	RPM1	8_1	Seita.4G250500	blight resistance protein SH20	Seita.2G294100	RPM1
Seita.3G107300	BPH14-1	3_2	Seita.8G039400	RPP13	8_1	Seita.4G250500	Disease resistant protein rga3	Seita.2G050400	Yr10
Seita.3G107300	BPH14-2	3_2	Seita.8G039400	Nitrate-induced NOI protein	8_1	Seita.4G214400	RPP13	Seita.2G103300	MLA6
Seita.3G241400	RPP13	3_3	Seita.8G039500	RGH1A	8_1	Seita.4G284200	RPP13	Seita.2G062100	RPM1
Seita.3G333300	RPM1	3_4	Seita.8G167300	Pi-b protein	8_10	Seita.7G250100	RGA4	Seita.2G176700	RGA2
Seita.3G393700	Rp1	3_6	Seita.8G166700	Pi-b protein	8_10	Seita.7G043100	RGA4	Seita.2G176700	RGA4
Seita.3G393700	rust resistance protein	3_6	Seita.8G166900	RPM1	8_10	Seita.7G306800	rp3	Seita.2G176700	Pi15
Seita.3G393700	Rp1-D	3_6	Seita.8G166900	Pib	8_10	Seita.7G306800	rp3-1	Seita.2G176700	Pi5-1
Seita.3G393500	Rp1	3_6	Seita.8G166900	Pi-b protein	8_10	Seita.7G014800	H0215A08.1	Seita.2G378800	RPM1
Seita.3G393500	Rp1-D	3_6	Seita.8G167500	Pi-b protein	8_10	Seita.7G187000	RPM1	Seita.2G057000	BPH14-1
Seita.3G396300	RPM1	3_7	Seita.8G167100	RPM1	8_10	Seita.7G311300	RGA1	Seita.2G057000	BPH14-2
Seita.3G395700	RPM1	3_7	Seita.8G167100	Pib	8_10	Seita.7G311300	RPP13	Seita.2G076700	Nbs1-Pi2
Seita.3G395700	RPP13	3_7	Seita.8G167100	Pi-b protein	8_10	Seita.7G044200	RGA4	Seita.2G078700	RPM1
Seita.3G396100	RGA2	3_7	Seita.8G182600	RGH1A	8_11	Seita.7G060600	RGA2	Seita.2G078700	MLA6
Seita.3G396100	NBS-LRR disease resistance protein homologue	3_7	Seita.8G181000	RPM1	8_11	Seita.7G288400	RGA1	Seita.2G075000	RPM1
Seita.3G400300	RGA1	3_8	Seita.8G181000	LRR14	8_11	Seita.7G288400	RPP13	Seita.2G075000	MLA6
Seita.3G400300	BPH14-1	3_8	Seita.8G183100	RPM1	8_11	Seita.6G220900	Pi-b protein	Seita.2G009000	MLA6
Seita.3G400300	powdery mildew resistance protein PM3b	3_8	Seita.8G183100	LRR14	8_11	Seita.6G232200	RGA1	Seita.2G011700	Nitrate-induced NOI protein
Seita.3G400200	Pm3b	3_8	Seita.8G181900	RPP13	8_11	Seita.6G232200	NBS3-RDG2A	Seita.2G178600	blight resistance protein SH20
Seita.3G400200	powdery mildew resistance protein PM3b	3_8	Seita.8G181900	Pib	8_11	Seita.6G232200	RDG2A	Seita.2G178600	Pi5-2
Seita.3G406200	Pm3b	3_9	Seita.8G184900	YNR1	8_14	Seita.6G235800	RGA1	Seita.2G246400	putative ATPase
Seita.3G406200	powdery mildew resistance protein PM3b	3_9	Seita.8G184900	YNR2	8_14	Seita.6G235800	NBS3-RDG2A	Seita.2G246400	RPM1
Seita.3G406300	Pm3b	3_9	Seita.8G184900	YNR3	8_14	Seita.6G021300	Lr21	Seita.2G246400	PPR1
Seita.3G406300	powdery mildew resistance protein PM3b	3_9	Seita.8G184900	YNR4	8_14	Seita.6G021300	rust resistance protein Rp1-dp8-like	Seita.2G246400	RXO1
Seita.4G126400	Nbs1-ON	4_1	Seita.8G184900	YNR5	8_14	Seita.6G092200	RPM1	Seita.2G055500	BPH14-1
Seita.4G126400	Nbs3-OP	4_1	Seita.8G192200	Yr10/Mla1	8_15	Seita.6G092200	RPP13	Seita.2G055500	BPH14-2
Seita.4G126400	Nbs7-75	4_1	Seita.8G191900	Yr10/Mla1	8_15	Seita.6G143000	MLA1	Seita.9G224800	RGA3
Seita.4G126400	Nbs1-Pi2	4_1	Seita.8G199600	Yr10/Mla1	8_16	Seita.1G011600	RGA-1	Seita.9G392900	RGA4
Seita.4G126400	Pi9	4_1	Seita.8G199500	Yr10/Mla1	8_16	Seita.1G011100	RGA-1	Seita.9G392900	XA1
Seita.4G126900	Nbs1-Pi2	4_1	Seita.8G201100	RGA4	8_17	Seita.1G053100	RPM1	Seita.9G296900	blight resistance protein RGA4
Seita.4G127000	NBA5	4_1	Seita.8G200400	Yr10/Mla1	8_17	Seita.1G053100	RPP13	Seita.9G296900	blight resistance protein SH20
Seita.4G244000	RPM1	4_2	Seita.8G200100	Yr10/Mla1	8_17	Seita.1G006400	RPP13	Seita.9G296900	blight resistance protein T118
Seita.4G243900	RPM1	4_2	Seita.8G050000	NBS-LRR disease resistance protein homologue	8_2	Seita.1G006400	Yr10	Seita.9G296900	RGA3
Seita.4G244400	RPM1	4_2	Seita.8G049900	RPH8A	8_2	Seita.1G072100	RPM1	Seita.9G298900	RPM1
Seita.5G230900	RPM1	5_1	Seita.8G049900	RPM1	8_2	Seita.1G072100	RPP13	Seita.9G466500	RPS2
Seita.6G014600	RPM1	6_1	Seita.8G049900	RPP13	8_2	Seita.1G191600	RPM1	Seita.9G419800	disease resistance protein At3g14460
Seita.6G014600	RGH1A	6_1	Seita.8G049900	RPP8	8_2	Seita.1G191600	RPP13	Seita.9G549300	RPP13
Seita.6G014500	RPM1	6_1	Seita.8G049900	Nitrate-induced NOI protein	8_2	Seita.1G010400	RGA-1	Seita.9G549300	blight resistance protein RGA3
Seita.6G014500	RGH1A	6_1	Seita.8G242600	Pm3b	8_20	Seita.J025700	RGA2	Seita.9G549300	RGA4
Seita.6G014800	RPM1	6_1	Seita.8G243100	RPM1	8_20	Seita.J025700	B0809H07.6	Seita.9G182800	RGA4
Seita.6G014800	RGH1A	6_1	Seita.8G242800	Pm3b	8_20	Seita.J025700	XA1		
Seita.6G014700	RPM1	6_1	Seita.8G249300	RGA2	8_21	Seita.3G274400	RPP13		
Seita.6G014700	RGH1A	6_1	Seita.8G249300	RGA4	8_21	Seita.3G388700	MLA1		
Seita.6G017100	Nbs1-ON	6_2	Seita.8G249100	RGA2	8_21	Seita.3G388700	Pi36		
Seita.6G017100	Nbs1-Pi2	6_2	Seita.8G249100	RGA4	8_21	Seita.3G338500	RPM1		
Seita.6G017500	NBS-LRR disease resistance protein homologue	6_2	Seita.8G064900	RPM1	8_3	Seita.3G317600	RPM1		
Seita.6G023600	RPM1	6_3	Seita.8G064400	RPM1	8_3	Seita.3G342000	RGC1B		
Seita.6G023600	RPP13	6_3	Seita.8G064700	RPM1	8_3	Seita.3G350300	RPM1		
Seita.6G023600	RGA1	6_3	Seita.8G064300	RPM1	8_3	Seita.3G278200	RGA3		
Seita.6G023500	RPM1	6_3	Seita.8G064300	LRR14	8_3	Seita.3G278200	putative blight resistance protein		
Seita.6G023500	RPP13	6_3	Seita.8G087200	MLA1	8_4	Seita.3G402500	powdery mildew resistance protein PM3b		
Seita.6G229200	RPM1	6_4	Seita.8G088100	RPM1	8_5	Seita.3G402500	Pm3b		
Seita.6G229200	Pib	6_4	Seita.8G088300	RPM1	8_5	Seita.3G221500	RPM1		
Seita.6G229200	Pi-b protein	6_4	Seita.8G088300	RPP8	8_5	Seita.8G184000	YNR1		
Seita.6G229600	Pi-b protein	6_4	Seita.8G089100	Nitrate-induced NOI protein	8_6	Seita.8G184000	YNR2		
Seita.6G229700	RPM1	6_4	Seita.8G088900	RPM1	8_6	Seita.8G184000	YNR3		
Seita.6G229700	Pi-b protein	6_4	Seita.8G089500	RPM1	8_6	Seita.8G184000	YNR4		
Seita.6G229300	Pi-b protein	6_4	Seita.8G089500	RPP13	8_6	Seita.8G184000	YNR5		
Seita.6G233300	RGA1	6_5	Seita.8G090100	RPM1	8_7	Seita.8G155500	RGA4		
Seita.6G233400	RGA1	6_5	Seita.8G090100	RPP13	8_7	Seita.8G202300	RPM1		
Seita.6G233400	NBS3-RDG2A	6_5	Seita.8G124300	RPM1	8_8	Seita.8G244000	Pm3b		
Seita.6G233400	RDG2A	6_5	Seita.8G162700	RGA4	8_9	Seita.8G183400	RPM1		
Seita.6G232800	RGA1	6_5	Seita.8G162600	RGA4	8_9	Seita.8G183400	LRR14		
Seita.6G232800	NBS3-RDG2A	6_5	Seita.9G374900	RGC1B	9_1	Seita.8G133400	Yr10		
Seita.6G232800	RDG2A	6_5	Seita.9G375200	RGC1B	9_1	Seita.8G048800	RGA4		

## 2 Experimental design, materials and methods

Exon and intron locations for the 242 identified R-genes were accessed from the Ensembl Genomes database [2] and uploaded to the Gene Structure Display Server [3] in Browser Extensible Data (BED) format to generate the visual display of structure. InterProScan and GO annotations [4], [5], also from the Ensemble Genomes Biomart, were downloaded and summarized in tabular format. Chromosome sequences for foxtail millet, rice, and barley genomes were uploaded in FASTA format to SyMAP version 4.2 [6] along with General Feature Format 3 (GFF3) annotation files. Using the 2D Chromosome Explorer function within SyMAP, figures were generated comparing chromosomes. Chromosomal locations for each of the R-genes of foxtail millet, rice, and barley were uploaded to the program R [7] to generate a density plot visualization. *S. bicolor* and *P. virgatum* R-genes [8], [9] were compiled with *S. italica* and *A. thaliana* sequences for the construction of a phylogenetic tree. Nucleotide-Binding, Apoptosis protease-activating factor-1, R-protein, *Caenorhabditis elegans* death-4 protein (NB-ARC) sequences were aligned within ClustalW [10] and a maximum-likelihood phylogenetic tree (100 bootstrap replicates) was generated in MEGA 7 [11], [12] using the JTT+G+F model, selected based upon a maximum-likelihood model test. This tree was edited in the Interactive Tree of Life server [13]. Using the South Dakota State University High Performance Computing Cluster and the PLAN server [14], BLAST results of the foxtail millet NB-ARC protein sequences were acquired and summarized in tabular format. *S. italica* accessions were updated to version 2.2 of the genome, available in Phytozome [15], [16].

## Figures and Tables

**Fig. S1:**